# Supplementary material for: Developmental delay and its associated factors among children under five years in urban slums of Nepal
Source: PLoS One. 2022 Feb 10;17(2):e0263105. doi: 10.1371/journal.pone.0263105 (PMC8830665; doi:10.1371/journal.pone.0263105)
Supplement: Attachment — Submitted filename: Response to Reviewers.docx [file pone.0263105.s003.docx]

We are grateful to all the reviewers for their time and effort to read and comment on our paper. Their comments are constructive and the paper has benefitted from it. Below we present the point-by-point response to the reviewer’s comments.

**Comments to the Author**

1. If the authors have adequately addressed your comments raised in a previous round of review and you feel that this manuscript is now acceptable for publication, you may indicate that here to bypass the “Comments to the Author” section, enter your conflict of interest statement in the “Confidential to Editor” section, and submit your "Accept" recommendation.

Reviewer #1: (No Response)

Reviewer #3: All comments have been addressed

**Thank you for your feedback.**

2. Is the manuscript technically sound, and do the data support the conclusions?

The manuscript must describe a technically sound piece of scientific research with data that supports the conclusions. Experiments must have been conducted rigorously, with appropriate controls, replication, and sample sizes. The conclusions must be drawn appropriately based on the data presented.

Reviewer #1: Yes

Reviewer #3: Partly

**We have worked to make the manuscript technically sound and revised the conclusions based on the findings.**

3. Has the statistical analysis been performed appropriately and rigorously?

Reviewer #1: Yes

Reviewer #3: No

**Thank you for your feedback. We have performed statistical analysis under proper guidance from expertise.**

4. Have the authors made all data underlying the findings in their manuscript fully available?

The [PLOS Data policy](http://www.plosone.org/static/policies.action#sharing) requires authors to make all data underlying the findings described in their manuscript fully available without restriction, with rare exception (please refer to the Data Availability Statement in the manuscript PDF file). The data should be provided as part of the manuscript or its supporting information, or deposited to a public repository. For example, in addition to summary statistics, the data points behind means, medians and variance measures should be available. If there are restrictions on publicly sharing data—e.g. participant privacy or use of data from a third party—those must be specified.

Reviewer #1: Yes

Reviewer #3: Yes

**Thank you for the feedback.**

5. Is the manuscript presented in an intelligible fashion and written in standard English?

PLOS ONE does not copyedit accepted manuscripts, so the language in submitted articles must be clear, correct, and unambiguous. Any typographical or grammatical errors should be corrected at revision, so please note any specific errors here.

Reviewer #1: Yes

Reviewer #3: Yes

**Thank you for the feedback.**

6. Review Comments to the Author

Please use the space provided to explain your answers to the questions above. You may also include additional comments for the author, including concerns about dual publication, research ethics, or publication ethics. (Please upload your review as an attachment if it exceeds 20,000 characters)

Reviewer #1: Dear Authors,

Thank you for your revision. There is one comment that is still of importance to reflect in your manuscript.

Please include the studies that have already done in Nepalese settings. Mentioning "Only few studies" will limit the significance/scientific merit of your manuscript. Briefly summaries those few studies related to developmental delays conducted in Nepal.

Thank you.

**Thank you for pointing it out. We have added brief summary of relevant studies as per your suggestion.**

In Introduction section:

Research estimating children at risk of development delay in overall aspects could be essential to highlight the need of interventions and policies targeting early childhood development [26]. Moreover, past studies have shown association between development outcomes and biological and anthropometrical [27], and nutritional factors [28] among children living in Nepal.

Reviewer #3: Dear authors,

Thank you so much for addressing my previous comments. Here are my comments regarding this revision.

1. Abstract: Your first and second sentence need some grammatical rephrasing. For example, you can omit unnecessary words like this. Children living in slum-like conditions of developing countries are at risk of exposure and threats that can adversely affect their ability to acquire full developmental potentials. I think you need to check this issue throughout your writing.

**Thank you for your feedback. We have worked on grammar and rephrased first and second sentence. Also, we have worked on grammatical errors and language used throughout the writing as much as possible.**

In Abstract section:

Children from low-resource settings are more likely to encounter those factors that adversely influence their ability to acquire developmental potential. This study was conducted to assess the developmental status and its associated factors among children under five years of slum areas of Butwal Sub Metropolitan City, Rupandehi, Nepal.

2. Methods: Is there any reason to select 30% of wards as states in "With that total number of wards remaining, 30% of the wards were selected by lottery method." or is it for convenience?

**We selected 30% of the wards randomly for convenience as the study had to be conducted using limited resources.**

3. Line 159-161. Could you provide reference for your classification for privileged and underprivileged ethnicity classification? "Attributes of family ethnicity like Dalit, Disadvantaged Janajati, Non-Dalit Disadvantaged Terai Caste, and Religious Minorities were added to form Underprivileged and Relatively Advantaged Janajati and Upper Caste Group were added to form Privileged."

**We have provided reference for the classification of privileged and underprivileged ethnicity as follows; however, we have also replaced it with Advantaged Group and Disadvantaged Group, respectively:**

Attributes of family caste-ethnicity like Dalit, Disadvantaged Janajati, Non-Dalit Disadvantaged Terai Caste, and Religious Minorities were added to form Disadvantaged Group and Relatively Advantaged Janajati and Upper Caste Group were added to form Advantaged Group [31].

4. Line 191-192: " Additionally, to measure the economic situation of household and socio-economic status, we included IWI related questions within the questionnaire. Could you provide reference for this?

**Thank you for your feedback. We have not provided any reference here because the International Wealth Index was already cited in the Study Parameters sub-section of Materials and Methods as follows.**

In Materials and Methods section:

Socio-demographic variables included of age in months and sex of children as stated by respondent, family ethnicity division (Dalit, Disadvantaged Janajati, Non-Dalit Disadvantaged, Terai Caste, Religious Minorities, Relatively Advantaged Janajati, Upper Caste Group) based on Health Management Information System of Nepal (HMIS) ethnicity classification [28], family type (nuclear, joint and extended), and socio-economic status based on International Wealth Index (IWI) classification [29] (extremely poor, poor, middle class, upper middle class, rich).

**However, we have cited it again as your feedback.**

In Materials and Methods section:

Additionally, to measure the economic situation of household and socio-economic status, we included IWI related questions within the questionnaire [29].

5. Line 206-208: While using logistic regression, how you decided your final model? Could you explain a bit in method section?

**Thank you for your feedback. We have provided explanation as follows.**

In Materials and Methods section:

Chi-square test and logistic regression were applied to determine the association between dependent and independent variables. All the statistical tests done were two-tailed and were considered statistically significant for a p-values<0.05 at 95% CI. While using logistics models, we adjusted for age of child, sex, family caste-ethnicity, family type, socio-economic status, mother’s age at birth of child, educational level, occupation, books available at home, language, parental smoking, parental alcoholism, birth weight, occurrence of infectious diseases in past 6 months, weight for height, weight for age and height for age.

6. Table: 1 to 3: I think you should not mention minimum and maximum like that, you can provide range as: logistic regression: correlation among independent variables, what is your final model?

**Thank you for your suggestion. We have removed minimum and maximum values included in the table.**

7. Line 247-255: While describing results, you can describe the association like this: On bivariate analysis, we found................" Otherwise, the meaning could be different.

On bivariate analysis, the age of children (p = 0.003) and their socio-economic status (p = 0.049) were associated with their developmental status. Status of availability of learning materials for children was associated (p <0.001) with the developmental status.

8. Line 280-281: "Findings related to prevalence in our study is much higher as compared to the prevalence rate of other studies done in developed nations." If you are writing in past tense then, be consistent. "was" instead of "is". Please check it throughout the piece.

**Thank you for pointing it out. We have revised it as follows:**

In Discussion section:

Findings related to prevalence in our study was much higher as compared to the prevalence rate of other studies done in developed nations [38,39].

9. Line 311-314: "Though many studies have shown there’s a strong link between the low birth weight of children with their developmental status [58,59]. However, children in our study didn’t have the disadvantage of being born with low birth weight, which is consistent with study done among Chinese [7] and Brazilian children [60]." This sentence require grammatical correction.

**Thank you for your feedback. We have revised the sentence as per your suggestion.**

In Discussion section:

Though many studies have shown there’s a strong link between the low birth weight of children with their developmental status [58,59]. However, children in our study had no consequence of being low weight at birth to their development, which is similar to the findings from the study done among Chinese [7] and Brazilian children [60].

10. Line 348-350: "Findings from the study suggest that there should be similar studies conducted among children living in slum-like conditions". How could you say this on the basis of your findings? Could you explain a bit?

**Thank you for pointing it out. We included this suggestion based on the finding about developmental delay which is high among children living in slum-like conditions in the present study. However, we have made some changes in the sentence as we wanted to indicate that interventions and research are needed to address such issues.**

In Conclusions section:

Findings from the study suggest that investigations need to focus on overall developmental aspects of early childhood development of children.

Overall, the revision seems nice, but you need to revise your writing in terms of reducing the redundancy and improving the grammatical issues.

**Thank you for your feedback. We have tried worked on reducing the redundancy and improving the grammatical errors.**
